# Supplementary material for: Case-Control Analysis of the Impact of Anemia on Quality of Life in Patients with Cancer: A Qca Study Analysis
Source: Cancers (Basel). 2021 May 21;13(11):2517. doi: 10.3390/cancers13112517 (PMC8196564; doi:10.3390/cancers13112517)
Supplement: Supplementary file 1 [file cancers-13-02517-s001.zip › cancers-1182378-s001.pdf]

cancers MDPI

Supplementary Material: Case-Control Analysis of the Impact of Anemia On Quality of Life in patients with Cancer: A Qca Study Analysis

Maria Barca-Hernando, Andres J. Muñoz-Martin, Eduardo Rios-Herranz, Ignacio Garcia-Escobar, Carmen Beato, Carme Font, Estefania Oncala-Sibajas, Alfonso Revuelta-Rodriguez, Maria Carmen Areses, Victor Rivas-Jimenez, Aitor Ballaz-Quincoces, Maria Angeles Moreno-Santos, Juan-Bosco Lopez-Saez, Iria Gallego-Gallego, Teresa Elias-Hernandez, Maria Isabel Asensio-Cruz, Leyre Chasco-Eguilaz, Gonzalo Garcia-Gonzalez, Purificacion Estevez-Garcia, Lucia Marin-Barrera, Remedios Otero-Candelera, Sergio Lopez-Ruz, Jorge Lima-Alvarez, Jose Maria Sanchez-Diaz, Macarena Real-Dominguez, Maria Carmen Borrego-Delgado, Samira Marin-Romero and Luis Jara-Palomares

Table S1. Recruitment per center.

Center	Type of Patient Included	Number of Patients
Hospital General Universitario Gregorio Marañón, Madrid, Spain	Controls	79
Hospital Virgen del Rocio, Sevilla, Spain	Cases and controls	64
Hospital Universitario Virgen de Valme, Sevilla, Spain.	Cases and controls	54
Hospital San Pedro Alcantara, Caceres, Spain	Controls	54
Hospital Universitario Virgen Macarena, Sevilla, Spain.	Cases	23
Hospital Clínic, Barcelona, Spain	Controls	23
Hospital Universitario Central de Asturias, Oviedo, Spain	Controls	22
Hospital de Orense, Orense, Spain.	Controls	15
Hospital de Jerez de la Frontera, Cádiz, Spain.	Controls	13
Hospital de Galdakao-Usansolo, Bizkaia, Spain	Cases	6
NISA Hospital, Sevilla, Spain.	Controls	5
H. U. Puerto Real, Cádiz, Spain	Cases and controls	4
Hospital Clínico San Carlos, Madrid.	Controls	3

Table S2. Marital status, education status, employment status, and gross income of the study patients.

Variable	Cases (Cancer with Anemia)	Controls (Cancer without Anemia)	Entire Cohort
Marital status (*n* = 328)			
Single, n (%)	10 (13.7%)	32 (12.5%)	42(12.8%)
Married, n (%)	52 (71.2%)	192 (75.3%)	244 (74.4%)
Separated, n (%)	1 (1.4%)	11 (4.3%)	12 (3.7%)
Divorced, n (%)	6 (8.2%)	7 (2.7%)	13 (4%)
Widowed, n (%)	4 (5.5%)	13 (5.1%)	17 (5.2%)
Education status (*n* = 305)			
No education, n (%)	2 (2.9%)	7 (3%)	9 (3.0%)
Primary, n (%)	26 (38.2%)	83 (35%)	109 (35.7%)
Secondary, n (%)	11 (16.2%)	28 (11.8%)	39 (12.8 %)
Vocational training, n (%)	9 (13.2%)	29 (12.2%)	38 (12.5%)
Middle school, n (%)	10 (14.7%)	27 (11.4%)	37 (12.1%)
University, n (%)	9 (13.2%)	51(21.5%)	60 (19.7%)
Other studies, n (%)	1 (1.5%)	12 (5.1%)	13 (4.3%)
Employment status (*n* = 298)			
Employed, n (%)	13 (20.3%)	55 (23.5%)	68 (22.8%)
Retired, n (%)	40 (62.5%)	132 (56.5%)	172 (57.7%)
Unemployed, n (%)	6 (9.4%)	25 (10.7%)	31 (10.4%)
Student, n (%)	1 (0.6%)	2 (0.9%)	3 (1%)
Homemaker, n (%)	4 (6.3%)	19 (8.1%)	23 (7.7%)
Other working conditions, n (%)	0 (0%)	1 (0.4%)	1 (0.3%)
Gross income (*n* = 198)			
19,999 Euros or less, n (%)	27 (61.4%)	102 (66.2%)	129 (65.2%)
20,000-39,999 Euros, n (%)	12 (27.3%)	41 (26.6%)	53 (26.8%)
40,000-59,999 Euros, n (%)	2 (4.5%)	7 (4.5%)	9 (4.5%)
60,000-99,999 Euros, n (%)	3 (6.8%)	3 (1.9%)	6 (3%)
More than 100,000 Euros, n (%)	0 (0%)	1 (0.6%)	1 (0.5%)

Table S3. Sex-related differences in terms of EORTC QLQ-c30, EQ-5D-3L questionnaire results in patients with anemia.

	**Men**					**Women**				
EORTC QLQ-C30 Functional scales	Anemia present	Anemia absent	Mean difference	ES (95% CI)	RE (95% CI)	Anemia present	Anemia absent	Mean difference	ES (95% CI)	RE (95% CI)
Global health status	42.5	59.6	−17.1 **	0.71 (0.4; 1.1)	−0.66 (−0.3; −1.0)	48.3	55.8	−7.5	0.85 (0.5; 1.3)	−0.29 (−0.6; 0.05)
Physical functioning	64.6	79.9	−15.3 **	0.63 (0.4; 1.0)	−0.63 (−0.3; −0.9)	69.6	77.6	−8 *	0.79 (0.5; 1.3)	−0.36 (−0.7; −0.01)
Role functioning	64.1	73.2	−9.1	0.78 (0.4; 1.2)	−0.27 (−0.6; 0,07)	55	69	−14 *	0.96 (0.5; 1.5)	−0.43 (−0.8; −0.07)
Emotional functioning	63.4	76.1	−12.7 **	0.5 * (0.3; 0.8)	−0.54 (−0.2; −0.9)	62.1	67.7	−5.6	0.84 (0.5; 1.4)	−0.20 (−0.5; 0.1)
Cognitive functioning	77.7	86.8	−9.1 *	0.6 * (0.3; 0.9)	−0.41 (−0.05; − 0.7)	83.3	80.6	2−7	1.02 (0.7; 1.9)	0.10 (−0.2; 0.4)
Social functioning	63.7	76.4	−12.7 *	0.6 * (0.3; 0.9)	−0.44 (−0.08; − 0.8)	64.7	68.0	−3.3	0.90 (0.5; 1.4)	−0.09 (−0,4; 0.25)
EORTC QLQ-C30 Symptoms scales	Anemia present	Anemia absent	Mean difference	ES (95% CI)	RE (95% CI)	Anemia present	Anemia absent	Mean difference	ES (95% CI)	RE (95% CI)
Fatigue	49	33.2	15.8 **	0.94 (0.5; 1.5)	0.56 (0.2; 0.9)	48.3	37.5	10.8 *	0.88 (0.5; 1.4)	0.37 (0.02; 0.7)
Nausea and vomiting	19.5	8.8	10.7 **	0.28 **(0.1; 0.4)	0.52 (0.1; 0.8)	21.7	10.6	11.1 **	0.42 **(0.2; 0.7)	0.51 (0.1; 0.9)
Pain	35.8	22.8	12.2 *	0.78 (0.4; 1.2)	0.42 (0.07; 0.7)	36	25.3	10.7*	0.80 (0.5; 1.3)	0.38 (0.03; 0.7)
Dyspnea	23	15.3	7.7	0.8 (0.5; 1.3)	0.28 (−0.07; 0.6)	20.4	13.7	6.7	0.68 (0.4; 1)	0.24 (−0.1; 0.6)
Insomnia	35	28.7	6.3	0.87 (0.5; 1.4)	0.28 (−0.1; 0.5)	33.3	32.1	1.2	0.91 (0.5; 1.5)	0.03 (-0.3; 0.4)
Appetite loss	39.1	21	18.1 **	0.46 **(0.2; 0.7)	0.59 (0.2; 0.9)	31.5	22.1	10.4	0.92 (0.5; 1.4)	0.3 (−0.03; 0.6)
Constipation	30.7	22.4	8.3	0.90 (0.5; 1.4)	0.27 (−0.08; 0.6)	23.0	22.2	0.8	0.86 (0.5; 1.4)	0.02 (−0.33; 0.4)
Diarrhea	20.1	13.3	6.8	0.55 **(0.3; 0.8)	0.26 (−0.09; 0.6)	22.7	15.9	6.8	0.60 *(0.3; 0.9)	0.24 (−0.1; 0.6)
Financial difficulties	17.5	18.5	−1	1.00 (0.6; 1.6)	−0.03 (−04; 0.3)	17.4	21.9	−4.5	1.20 (0.7; 2)	−0.15 (−0.5 ;0.2)
EQ-5D-3L	Anemia present	Anemia absent	Mean difference	ES (95% CI)	RE (95% CI)	Anemia present	Anemia absent	Mean difference	ES (95% CI)	RE (95% CI)
Index score	0.67	0.75	−0.08	0.68 (0.3; 1.1)	−0.24 (−0.6; 0.1)	0.64	0.7	0.06	1.14 (0.7; 1.8)	−0.19 (−0.5; 0.1)

Abbreviations: CI: confidence interval. *$p < 0.05$; ** $p < 0.001$. ES: effect size; RE: relative efficiency. EORTC QLQ-C30 functional scale: Higher scores reflect a better health status and the minimally important difference varies from 6 to 15 points; EORTC QLQ-C30 symptoms scale: Higher scores in the symptoms scale indicate a reduction in quality of life; EQ-5D-3L: Higher scores reflect a better health status and the minimally important difference on the EQ-5D questionnaire for patients with malignancies is 0.06–0.08 UI.

Table S4. Differences in the EORTC QLQ-C30 functional scales in patients with anemia according to tumor site.

		Global Health Status	Physical Functioning	Role Functioning	Emotional Functioning	Cognitive Functioning	Social Functioning
Gynecologic	Anemia present	33.3	57.7	38.9	47.3	63.9	52.8
	Anemia absent	43.8	80.5	69.2	68.0	84.6	74.0
	Mean difference	−20.5 *	−22.8 *	−30.3	−20.8	−20.7	−21.2
	Effect size (95% CI)	1.04 (0.2; 3.3)	0.54 (0.08; 1.6)	0.72 (0.1; 2.2)	0.40 (0.06; 1.2)	0.56 (0.09; 1.7)	0.80 (0.1; 2.5)
	Relative efficiency (95% CI)	−0.92 (−1.8; 0.02)	−1.01(−2.0; −0.05)	−0.88 (−1.8; 0.06)	−0.85 (−1.8; 0.09)	−0.90 (−1.8; 0.05)	−0.68 (−1.6; 0.26)
Lung	Anemia present	53.2	70.2	55.1	68.6	85.8	55.1
	Anemia absent	63.1	82.6	74.0	71.0	85.8	70.9
	Mean difference	−9.9	−12.4 *	−18.9 *1	−2.4	0.0	−15.8
	Effect size (95% CI)	0.5 (0.2; 1.1)	0.32 *(0.1; 0.7	0.55 (0.2; 1.2)	0.67 (0.2; 1.4)	1.46 (0.5; 3.2)	0.38 *(0.1; 0.8)
	Relative efficiency (95% CI)	−0.4 (−1.0; 0.2)	−0.6 (−1.2; −0.004)	−0.6 (−1.2; −0.01)	−0.1 (−0.7; 0.5)	0.004 (−0,6; 0.6)	
Digestive	Anemia present	45.6	61.0	55.3	57.4	84.2	68.4
	Anemia absent	59.0	81.5	76.0	76.0	83.6	77.7
	Mean difference	−13.4 *	−20.5 *	−20.7 *	−18.6 *	0.6	−9.4
	Effect size (95% CI)	0.55 (0.2; 1.0)	0.44 ** (0.2; 0.9)	0.69 (0.3; 1.3)	0.67 (0.3; 1.3)	2.14 (0.3; 1.3)	0.78 (0.3; 1.5)
	Relative efficiency (95% CI)	−0.52 (−1.0; −0.001)	−1.0 (−1.5; −0.5)	−0.7 (−1.2; −0.2)	−0.7 (−1.2; −0.2)	0.02 (−0.5; 0.5)	−0.33 (−0.8; 0.2)
Lymphomas	Anemia present	43.1	73.7	70.8	62.7	76.5	72.5
	Anemia absent	58.0	74.8	67.5	68.5	80.0	64.3
	Mean difference	−14.9	−1.1	3.3	−5.8	−3.5	8.2
	Effect size (95% CI)	0.65 (0.2; 1.4)	0.93 (0.4; 2.0)	1.21 (0.4; 2.7)	0.53 (0.2; 1.2)	0.50 (0.2; 1.1)	0.77 (0.3; 1.7)
	Relative efficiency (95% CI)	−0.55 (−1.1; 0.04)	−0.04 (−0.6; 0.5)	0.08 (−0.5;0.7)	−0.20 (−0.8;0.4)	−0.11 (−0.7;0.4)	0.24 (−0.3; 0.8)
Others	Anemia present	45.6	66.1	62.2	67.0	85.6	60.7
	Anemia absent	53.4	74.6	66.9	74.0	85.2	73.4
	Mean difference	−7.8	−8.5	−4.7	−7.0	−2.5	−12.7
	Effect size (95% CI)	1.25 (0.6; 2.4)	1.31 (0.6; 2.4)	1.07 (0.5; 2.0)	1.09 (0.5; 2.0)	1.07 (0.5; 2.0)	0.95 (1.5; 1.8)
	Relative efficiency (95% CI)	−0.28 (−0.7; 0.2)	−0.32 (−0.7; 0.1)	−0.13 (−0.6; 0.3)	−0.29 (−0.7; 0.2)	−0.11 (−0.6; 0.3)	−0.41 (−0.9; 0.06)

Abbreviations: CI: confidence interval. *$p < 0.05$; ** $p < 0.001$. EORTC QLQ-C30 functional scale: Higher scores reflect a better health status and the minimally important difference varies from 6 to 15 points

Table S5. Differences in the EORTC QLQ-C30 symptoms scales in patients with anemia according to tumor site.

**EORTC QLQ-C30 Symptoms scales**		**Fatigue**	**Nausea and Vomiting**	**Pain**	**Dyspnea**	**Insomnia**	**Appetite Loss**	**Constipation**	**Diarrheal**	**Financial Difficulties**
Gynecologic	Anemia present	72.2	30.5	47.2	40.0	40.0	61.1	11.1	38.9	44.4
	Anemia absent	38.0	12.9	28.4	12.8	32.0	23.4	28.0	38.9	25.3
	Mean difference	34.2 **	17.6	18.8	27.2*	8.0	37.6*	16.9	14.9	19.9
	Effect size (95% CI)	0.67 (0.1; 2.1)	0.28 (0.04; 0.9)	0.95 (0.15; 2.9)	2.82 (0.3; 9.5)	0.54 (0.06; 1.8)	1.0 (0.2; 3.4)	0.83 (0.1; 2.6)	0.32 (0.05; 1.0)	1.16 (0.2; 3.6)
	Relative efficiency (95% CI)	1.3 (0.3; 2.2)	0.7 (0.2; 1.6)	0.78 (0.1; 1.7)	1.1 (0.09; 2.2)	0.2 (−0.8; 1.2)	1.1 (0.1; 2.0)	−0.7 (−1.3; 0.3)	0.4 (−0.5; 1.3)	0.6 (−0.3; 1.6)
Lung	Anemia present	45.3	26.9	35.9	23.1	43.6	36.1	25.6	20.5	20.5
	Anemia absent	32.6	11.7	23.7	11.1	33.3	25.3	18.2	13.6	18.8
	Mean difference	12.7	15.2 *	12.1	12.0	10.2	10.8	7.4	6.9	1.7
	Effect size (95% CI)	0.67 (0.2; 1.5)	0.28 **(0.09; 0.6)	0.67 (0.2; 1.5)	0.80 (0.3; 1.8)	1.09 (0.4; 2.4)	0.54 (0.2; 1.2)	0.54 (0.2; 1.2)	0.55 (0.2; 1.2)	0.48 (0.2; 1.0)
	Relative efficiency (95% CI)	0.46 (−0.1; 1.0)	0.64 80.02; 1.2)	0.44 (−0.2; 1.0)	0.52 (−0.1; 1.4)	0.31 (−0.3; 0.9)	0.33 (−0.3; 1.0)	0.27 (−0.3; 0.9)	0.27 (−0.3; 0.9)	0.05 (−0.55; 0.6)
Digestive	Anemia present	49.7	21.3	36.8	24.5	29.8	36.8	27.7	24.5	14.0
	Anemia absent	33.3	7.6	22.3	10.7	29.4	18.6	17.2	15.9	21.7
	Mean difference	16.4 *	13.7 *	14.5 *	13.8*	0.4	18.2*	10.6	8.6	−7.7
	Effect size (95% CI)	0.95 (0.4; 1.8)	0.30 **(0.1; 0.6)	0.86 (0.4; 1.7)	0.37 *(0.1; 0.7)	1.48 (0.6; 2.9)	0.60 (0.3; 1.2)	1.2 (0.5; 2.4)	0.48 *(0.2; 0.9)	1.75 (0.7; 3.4)
	Relative efficiency (95% CI)	0.62 (0.1; 1.11)	0.68 (0.1; 1.12)	0.5 (−0.03; 1.1)	0.55 (0.03; 1.0)	0.01 (−0.5; 0.5)	0.60 (0.07; 1.1)	0.37 (−0.1; 0.9)	0.32 (−0.2; 0.8)	−0.26 (−0.8; 0.3)
Lymphomas	Anemia present	39.9	22.5	31.2	23.5	33.3	45.0	37.7	23.5	23.5
	Anemia absent	35.7	8.3	22.7	19.4	27.8	18.5	29.4	7.6	18.0
	Mean difference	4.1	14.2*	8.5	4.1	5.5	26.6**	8.3	15.9	5.5
	Effect size (95% CI)	1.28 (0.5; 2.8)	0.25 (0.09; 0.55)	0.79 (0.3; 1.8)	0.59 (0.2; 1.3)	0.55 (0.2; 1.2)	0.40 *(0.1; 0.9)	0.84 (0.3; 1.9)	0.24 **(0.09; 0.5)	0.85 (0.3; 1.9)
	Relative efficiency (95% CI)	0.13 (−0.5; 0.7)	0.61 (0.09; 1.2)	0.24 (−0.4; 0.8)	0.13 (−0.4; 0.7)	0.16 (−0.4; 0.7)	0.85 (0.2; 1.4)	0.22 (−0.4; 0.8)	0.69 (0.08; 1.3)	0.18 (−0.4; 0.8)
Other sites	Anemia present	52.1	15.4	35.2	16.7	33.3	26.9	26.6	16.6	9.7
	Anemia absent	37.2	8.2	24.1	18.5	32.3	20.6	21.5	12.4	17.5
	Mean difference	14.9 *	7.2	11.1	−1.8	1.0	6.3	5.1	4.2	−7.8
	Effect size (95% CI)	1.15 (0.5; 2.1)	0.53 *(0.3; 0.9)	0.85 (0.4; 1.6)	1.21 (0.6; 2.2)	0.92 (0.4; 1.7)	0.81 (0.4; 1.5)	0.86 (0.4; 1.6)	0.74 (0.3; 1.4)	2.84 (1.3; 5.4)
	Relative efficiency (95% CI)	0.51 (0.04; 0.9)	0.38 (−0.08; 0.08)	0.38 (−0.08;0.8)	0.24 (−0.4; 0.8)	0.03 (−0.4; 0.5)	0.20 (−0.2; 0.7)	0.17 (−0.3; 0.6)	0.16 (−0.3; 0.6)	−0.28 (−0.8; 0.2)

Abbreviations: CI: confidence interval. *$p < 0.05$; ** $p < 0.001$. EORTC QLQ-C30 symptoms scale: Higher scores in the symptoms scale indicate a reduction in quality of life; the minimally important difference varies from 6 to 15 points.

Table S6. Differences in the EQ-5D-3L results in patients with anemia according to tumor site.

**EQ-5D-3L**		**Index Score**
Gynecologic	Anemia present	0.35
	Anemia absent	0.70
	Mean difference	−0.35 *
	Effect size	0.87
	(95% CI)	(0.1; 2.9)
	Relative efficiency	−1.17
	(95% CI)	(−2–2; −0.1)
Lung	Anemia present	0.60
	Anemia absent	0.79
	Mean difference	−0.18 *
	Effect size	0.28 *
	(95% CI)	(0.09; 0.6)
	Relative efficiency	−0.71
	(95% CI)	(−1.3; −0.07)
Digestive	Anemia present	0.69
	Anemia absent	0.78
	Mean difference	−0.06
	Effect size	0.63
	(95% CI)	(0.3; 1.3)
	Relative efficiency	−0.23
	(95% CI)	(−0.8; −0.3)
Lymphoma	Anemia present	0.71
	Anemia absent	0.72
	Mean difference	−0.00
	Effect size	0.92
	(95% CI)	(0.4; 2.0)
	Relative efficiency	−0.005
	(95% CI)	(−0.6 −0.6)
Other sites	Anemia present	0.64
	Anemia absent	0.67
	Mean difference	−0.03
	Effect size	1.92
	(95% CI)	(0.9; 3.6)
	Relative efficiency	−0.0.6
	(95% CI)	(−0.5; 0.4)

Abbreviation: CI: confidence interval. **p* <0.05; ** *p* <0.001. EQ-5D-3L: Higher scores reflect a better health status and the minimally important difference on the EQ-5D questionnaire for patients with malignancies is 0.06–0.08 UI.

Table. S7. Differences in terms of QLQ-c30 functional and symptom scales scores in patients with anemia, comparing patients with and without VTE.

QLQ-c30 Functional Scales	Anemia and VTE Present	Anemia and VTE Absent	Mean Difference	Effect Size (95% CI)	Relative Efficiency (95% CI)
Global health status	43.09	47.39	−4.30	0.95 (0.51;1.80)	−0.15 (−0.59; 0.28)
Physical functioning	60.19	72.34	−12.15 *	1.00 (0.54;1.92)	−0.47 (−0.92; −0.03)
Role functioning	49.52	66.66	−17.14 *	0.90 (0.48;1.72)	−0.51 (−0.92; −0.06)
Emotional functioning	64.52	61.45	3.07	0.97 (0.52; 1.87)	0.10 (−0.33; 0.54)
Cognitive functioning	83.80	78.47	5.33	0.71 (0.38; 1.36)	0.21 (−0.22; 0.65)
Social functioning	65.23	63.54	1.69	1.19 (0.64; 2.28)	0.04 (−0.39; 0.49)
QLQ−c30 Symptoms scales					
Fatigue	50.95	47.05	3.90	1.45 (0.78; 2.78)	0.13 (−0.30; 0.57)
Nausea and vomiting	18.13	22.44	−4.31	0.61 (0.33; 1.18)	−0.14 (−0.58; 0.30)
Pain	39.04	32.98	6.06	0.74 (0.40; 1.43)	0.19 (−0.24; 0.64)
Dyspnea	26.66	18.05	8.61	1.00 (0.54; 1.92)	0.28 (−0.15; 0.73)
Insomnia	31.37	36.05	−4.68	0.95 (0.51; 1.82)	−0.13 (−0.58; 0.31)
Appetite Loss	24.50	43.53	−19.03 *	0.91 (0.49; 1.75)	−0.52 (−0.97; −0.07)
Constipation	31.37	23.18	8.19	1.53 (0.81; 2.96)	0.25 (−0.19; 0.70)
Diarrhea	15.68	25.69	−10.01	1.28 (0.68; 2.47)	−0.30 (−0.75; 0.14)
Financial difficulties	17.17	17.73	−0.56	0.91 (0.49; 1.75)	−0.02 (−0.46; 0.42)
EQ−5D-3L					
Index score	0.53	0.75	−0.22 **	1.92 (1.02; 3.74)	−0.65 (−1.11; −0.18)

Abbreviations: CI: confidence interval. *$p < 0.05$; ** $p < 0.001$. EORTC QLQ-C30 functional scale: Higher scores reflect a better health status and the minimally important difference varies from 6 to 15 points. EORTC QLQ-C30 symptoms scale: Higher scores in the symptoms scale indicate a reduction in quality of life. EQ-5D-3L: Higher scores reflect a better health status and the minimally important difference on the EQ-5D questionnaire for patients with malignancies is 0.06–0.08 UI.5D-3L: Higher scores reflect a better health status and the minimally important difference on the EQ-5D questionnaire for patients with malignancies is 0.06–0.08 UI.
